# The double suprascapular foramen: unique anatomical variation and the new hypothesis of its formation

**DOI:** 10.1007/s00256-012-1460-z

**Published:** 2012-06-22

**Authors:** Michał Polguj, Michał Podgórski, Kazimierz Jędrzejewski, Mirosław Topol

**Affiliations:** 1 Department of Angiology, Chair of Anatomy, Medical University of Łódź, 90-136 Narutowicza 60, Łódź, Poland; 2 Department of Normal and Clinical Anatomy, Chair of Anatomy, Medical University of Łódź, Łódź, Poland

**Keywords:** Suprascapular foramen double, Anatomical variation, Suprascapular nerve entrapment

## Abstract

A unique anatomical variation of the suprascapular notch was discovered in one scapula from 610 analyzed by three-dimensional CT reconstruction. Two bony bridges were found, converting it into a double suprascapular foramen, in the left upper extremity of an 56-year-old Caucasian female. This variation might be a risk factor for suprascapular nerve entrapment. Suprascapular nerve running through inferior suprascapular foramen was discovered. Suprascapular vessels passed through superior suprascapular foramen (artery lay medially and vein laterally). A new hypothesis of double suprascapular foramen formation (mechanism of creation) is presented based on recent anatomical findings (e.g., the discovery in 2002 of the anterior coracoscapular ligament). Knowledge of the anatomical variations described in this study should be helpful in arthroscopic and open procedures at the suprascapular region and also confirms the safety of operative decompression for the suprascapular nerve.

## Introduction

The suprascapular notch (SSN) is the site where the suprascapular nerve (SN), accompanied by its associated vein, traverses the upper border of the scapulae under the superior transverse scapular ligament (STSL). The corresponding artery runs over the ligament. This region is the most common location of suprascapular nerve injury and compression [1–4]. One of the most important predepositing factors of this neuropathy is an ossified superior transverse scapular ligament [2, 5–8]. In such cases, a suprascapular foramen is formed. Its frequency depends on the population type. In the scientific literature, there have only been two described cases of a double suprascapular foramen [9, 10]. As it is a condition that decreases the area of the SSN, it might be also considered as a risk factor for SN neuropathy.

It has been hypothesized that double suprascapular foramen formation was associated with ossification of the bifid STSL [10]. However, a new possible mechanism for the creation of this anatomical variation was suggested by the discovery in 2002 of the anterior coracoscapular ligament (ACSL) by Avery et al. [11]. To our knowledge, the double suprascapular foramen described in this study has not been reported previously in the European population. This case also presents several hypotheses of its formation based on the latest anatomical findings, and the first report of a potentially ossified ACSL.

## General study

CT scans of 610 shoulders were retrospectively analyzed in 305 randomized patients who were being investigated as part of standard CT chest protocol between May 2008 and December 2011 for a disease of the lungs or cardiovascular system. The patients with metastases to bone were excluded from the studied group. The aim of the study was analysis of variations of the suprascapular notch in a Polish population. The research project and procedures were approved by the Bioethics Commission of the Medical University of Lodz. Dual-phase helical CT was performed with a 32-row MDCT scanner (Toshiba Aquilion 32; Toshiba Medical System). All scapulae were analyzed with post-processing tools; MPR and MIP images were obtained on coronal and sagittal planes, as well as coronal curved MIP images, and three-dimensional VR were acquired. Reconstructed images were evaluated in consensus by two independent scientists with 5 years of experience. The measurements of the three-dimensional CT reconstructions were performed using Vitrea 2 system software (Vital Images, Plymouth, MN, USA).

In the studied group, we distinguished several variations of the suprascapular notch. Seventy-eight scapulas had a discrete notch. In 149 scapulas, maximal depth of the suprascapular notch was longer than its maximal width. Thirteen cases had equal both dimensions. In 337 scapulas, the maximal width of the suprascapular notch was longer than its maximal depth. Bony foramen (corresponding to completely ossified superior transverse suprascapular ligament) was present in 32 cases.

## Case report

**Fig. 1:**
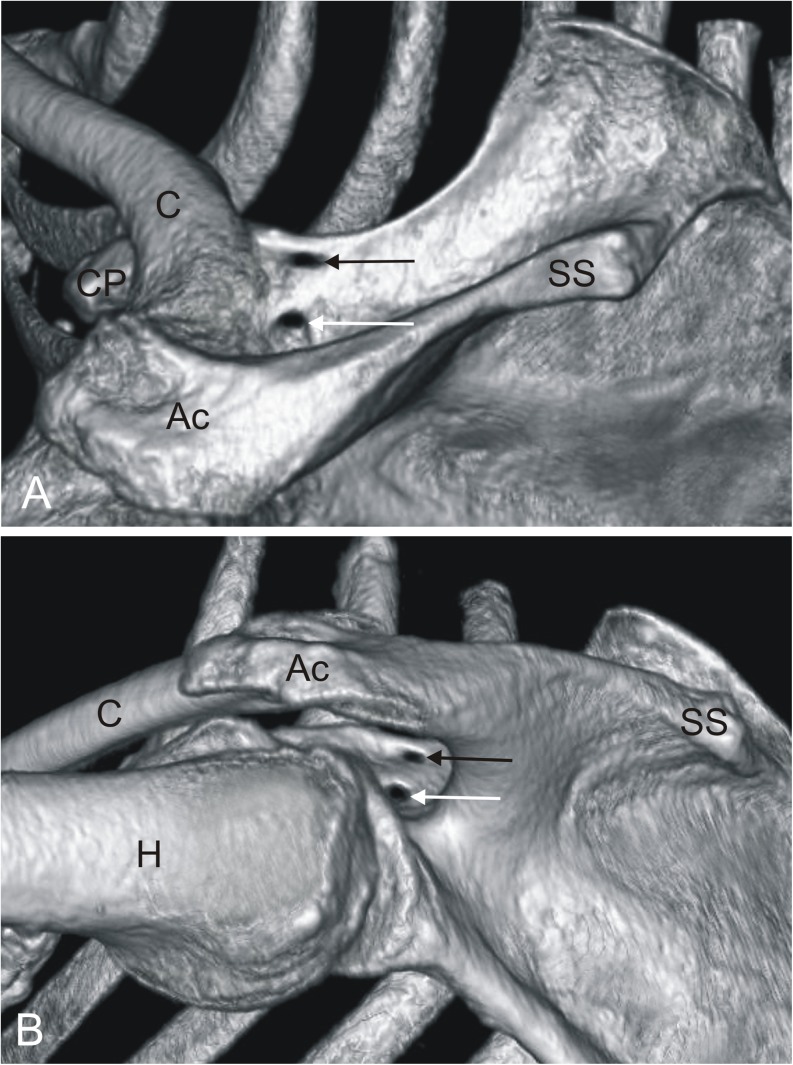
Three-dimensional volume rendering (VR) MDCT demonstrating double suprascapular foramen: **a** supero-posterior view; **b** infero-posterior view. → *black arrow* superior suprascapular foramen, → *white arrow* inferior suprascapular foramen, *Ac* acromion, *C* clavicle, *CP* coracoid process, *H* humerus, *SS* scapular spine

The unique anatomical variation of the suprascapular notch where two bony bridges convert it into a double suprascapular foramen was discovered in one of the 610 analyzed scapulae (Fig. 1). The finding was present in the left upper extremity of a 56-year-old Caucasian female. The reason for her hospitalization was suspicion of a pulmonary embolism. The patient had no symptoms of suprascapular nerve entrapment syndrome. The superior bridge was 13.7 mm long, and its width in the proximal, middle, and distal portions were respectively, 5.1 mm, 3.8 mm, and 6.2 mm. The inferior osseous bridge was 9.8 mm long, 12.3 mm wide in the proximal portion, 9.9 mm wide in the middle portion and 10.3 mm wide in the distal portion. The area of the upper foramen was 22.6 mm^2^ and the lower one, 14.9 mm^2^.

**Fig. 2:**
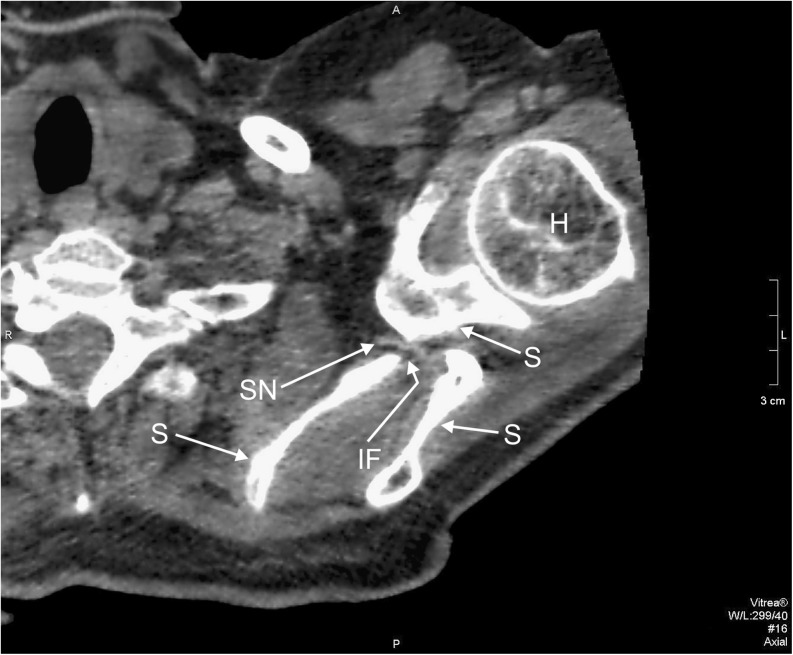
Dual-phase helical CT, transverse scan on the level of the inferior suprascapular foramen: *H* humerus, *IF* inferior suprascapular foramen, *SN* suprascapular nerve, *S* scapula

**Fig. 3:**
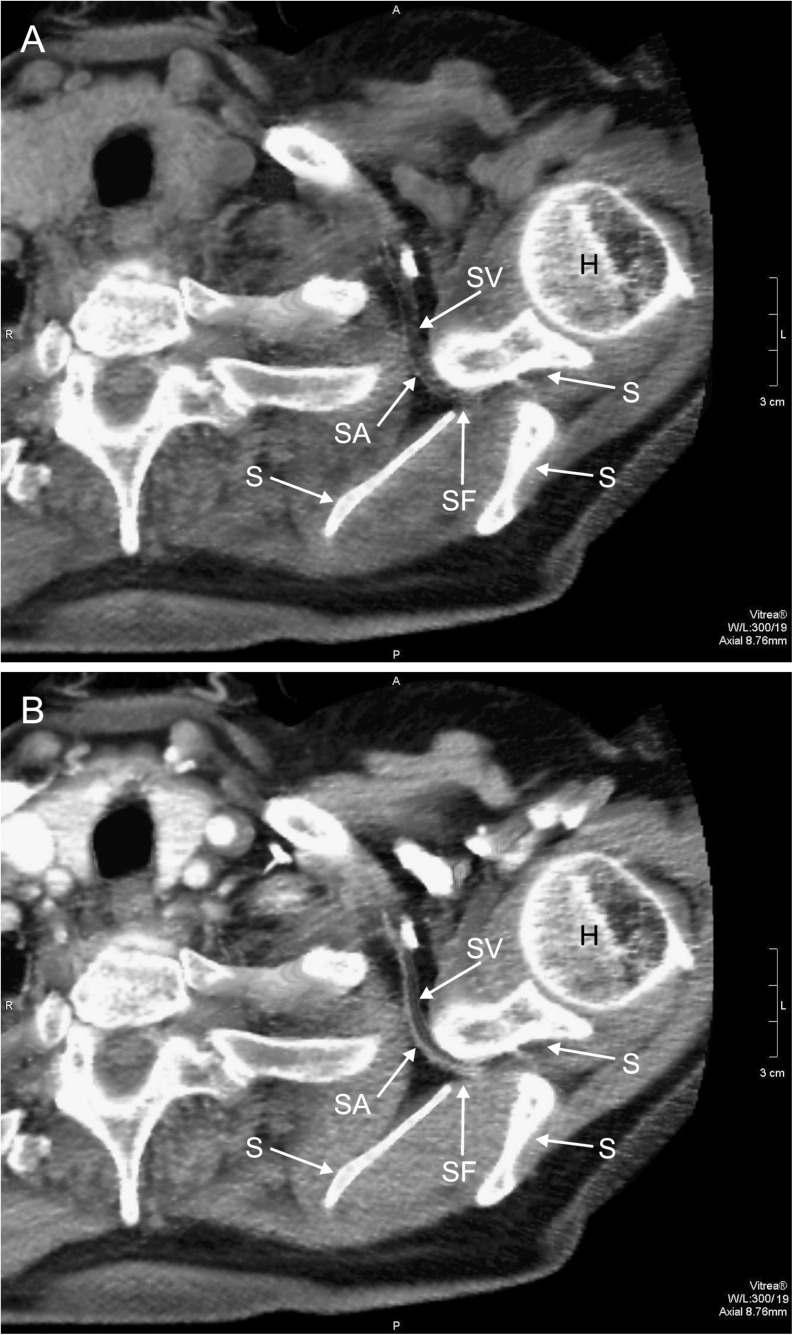
Dual-phase helical CT, transverse scan on the level of the superior suprascapular foramen: *A* without contrast material, *B* after injection of contrast medium. *H* humerus, *SF* superior suprascapular foramen, *SA* suprascapular artery, *SV* suprascapular vein, *S* scapula

In the dual-phase helical CT investigation, topography of the suprascapular nerve and vessels was evaluated. The nerve traveled through the inferior suprascapular foramen (Fig. 2), and vessels ran by the superior foramen (artery medially, vein laterally) (Fig. 3). Vessels were distinguished by using two phases: first without contrast material (Fig. 3a) and second after a mechanical injection of nonionic iodinated contrast medium (Ultravist 370, Bayer Schering Pharma AG, Germany) (Fig. 3b).

## Discussion

A double suprascapular foramen has been described in the literature only twice [9, 10]. Hrdlica [9] described such a case in the scapula of a Caucasian male in 1942 and Wang et al. [10] presented this type of notch in a Chinese individual. The latter proposed ossification of a bifid superior transverse scapular ligament as an explanation. The diversity of STSL anatomy drew researchers' attention because unlike the notch type variation, it has been associated with suprascapular nerve injury or compression. Calcification of the ligament has been determined to be the one of factors increasing the danger of suprascapular neuropathy [12]. The incidence of complete ossification of the STSL depends on population and has been found to vary from 3 to 12.5 % [2, 3, 5, 7–10, 13–22] (Table 1). A familial case of the calcification of the STSL causing entrapment neuropathy of the SN affecting both father and son has also been described, suggesting that the ossification may have a genetic basis [6].

**Table 1:** Frequency of ossifications of the superior transverse scapular ligament in different populations

Researcher	Country	Ossification (%)		Number of studied specimens (*n*)
		Total	Partial	
Olivier [15]	France	5	-	100
Vallois [21, 22]		6.5	-	146
	Italy	6.1	-	152
Bayramoglu et al. [5]	Turkey	12.5	-	32
Natsis et al. [14]	Germany	7.3	-	423
Polguj et al. [8, 16]	Poland	7	23.3	86
Edelson [7]	USA	3.7	18	1,000
Tubbs et al. [20]		3.7	-	120
Rengachary et al. [2, 17]		4	-	211
Dunkelgrun et al. [13]		5	12	623
Ticker et al. [19]		5	81	79
Sinkeet et al. [18]	Kenya	3	-	138
Wang et al. [10]	China	4.08	-	295
Presented study	Poland	5.2	-	610

**Fig. 4:**
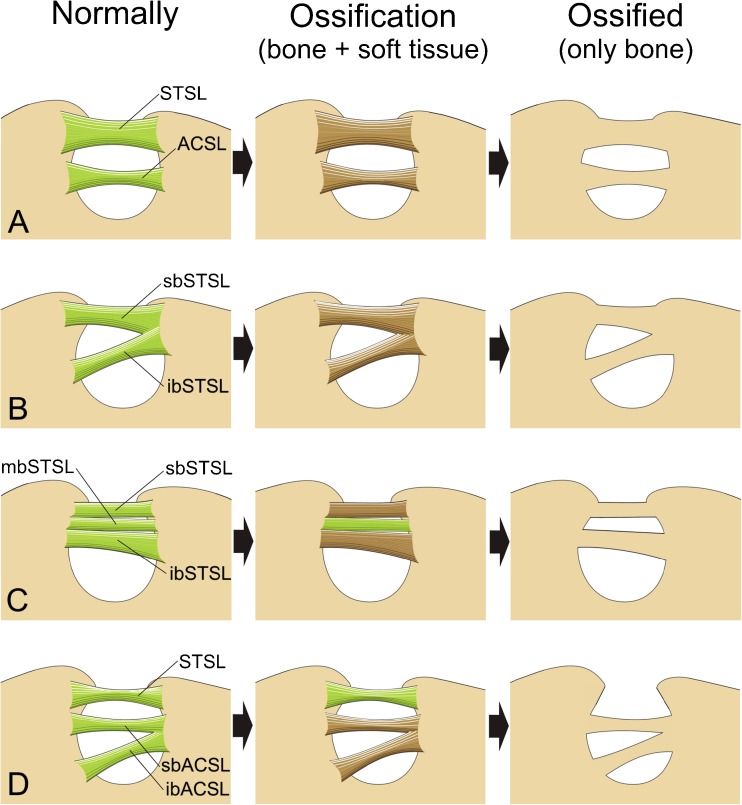
Schematic arrangements demonstrating hypotheses of double suprascapular notch formation: **a** first hypothesis, **b** second hypothesis, **c** third hypothesis, **d** fourth hypothesis. *STSL* superior transverse scapular ligament, *sbSTSL* superior band of superior transverse scapular ligament, *mbSTSL* middle band of superior transverse scapular ligament, *ibSTSL* inferior band of superior transverse scapular ligament, *ACSL* anterior coracoscapular ligament, *sbACSL* superior band of anterior coracoscapular ligament, *ibACSL* inferior band of anterior coracoscapular ligament

We propose four potential hypotheses of double suprascapular foramen formation based on the most recent anatomical findings (Fig. 4). The first hypothesis is based on the assumption that the ossification of the single bundle STSL would create the upper bony bridge of the double suprascapular foramen. The lower bridge would then be created by the osseous transformation of the anterior coracoscapular ligament (Fig. 4a). This hypothesis is supported by the parallel course of the lower bony bridge and distinct attachments to the SSN margins. The ACSL was first described by Avery et al. [11] and it is a fibrous band extending along the anterior aspect of the SSN, just below the STSL. Its proximal and distal attachments insert separately on the middle and lateral border of the SSN and can be oriented parallel or obliquely to the STSL. However, no previous cases of ACSL ossification have been reported.

Due to the significant narrowing of the functional opening of the suprascapular foramen, the ACSL has an apparent potential impact on the occurrence of suprascapular nerve neuropathy [5, 11]. In our opinion, the ossification of the ACSL, accompanied by the ossified STSL, can increase that risk because of the higher potential for nerve irritation by the bony margins of the foramen and the lack of elasticity that the ACSL normally demonstrates. Although it is impossible to determine the course of the SN in presented case, the area of each of these bony foramina was highly reduced in comparison to the potential area of the notch without a lower bony bridge. The frequency of the ACSL varied from 18.8 % [5] to 60 % [11] of cases. Also, Bayramoğlu et al. [5] confirmed its presence as an additional etiological factor of the condition. In our opinion, it is the most probable hypothesis.

The second potential mechanism explaining the formation of a double suprascapular foramen is calcification of the bifid STSL. In such a case, the ligament would have two bands (superior and inferior) that are separately fixed on the border of the suprascapular notch. Both parts of this bifid STSL travel independently, one below the other, but have a common opposite attachment. All parts of the ligament have to be simultaneously and completely ossified (Fig. 4b). Recent literature has presented a few cases of bifid superior transverse scapular ligaments [5, 19, 23]. Its frequency was found to be from 3.3 % [23] to 15.6 % [5].

The third hypothesis explaining the variation described in this article would be a partial ossification of the trifid superior transverse scapular ligament. This type of STSL has three parts: the superior band, the middle band, and the inferior band. Only in the case of ossification of the superior and inferior bands is a double suprascapular foramen formed (Fig. 4c). Recent literature has shown only one case of trifid STSL [19]. However, the ligament described by Ticker et al. [19] had the middle band ossified, making this hypothesis rather doubtful.

The fourth potential mechanism explaining double suprascapular foramen formation is the calcification of the bifid anterior coracoscapular ligament (Fig. 4d). However, this seems less likely because in such an anatomical variant, there should be a notch above the two bony foramina. To our knowledge, such a case has never been published.

In the presented study, the suprascapular vein and artery run through the superior suprascapular foramen (Fig. 3). In 2012, Yang et al. [24] described topography of the suprascapular nerve, artery, and vein at the suprascapular notch (cadavers from Korean population). According to their study, in 11 from 103 dissected shoulders (10.9 %), suprascapular vessels traveled below the superior transverse scapular ligament (type III of their classification). However, contrary to this description in the research of Yücesoy et al. [25], suprascapular vessels always traveled below STSL. The suprascapular artery-vein complex was visualized in a total of 43 from 50 (86 %) Turkish volunteers by color Doppler ultrasound.

Taking into consideration suprascapular nerve topography, our first hypothesis of double suprascapular foramen formation confirms Avery et al. [11] study. Scientists discovered the presence of the ACSL in 60 % of 54 dissected shoulders. In their examination, the suprascapular nerve always was found to pass below the ACSL, thus bringing the nerve in close contact to the bony floor of the suprascapular notch. Researchers have highlighted its role in the narrowing of the suprascapular foramen, which can potentially increase the risk of nerve entrapment. Our description of the suprascapular nerve course in the inferior suprascapular foramen is similar to Avery et al. [11] study. Also Bayramoğlu et al. [5] considered ACSL as an additional etiological factor of the condition.

The separation of the vascular bundle from the nerve has pragmatic implications to the suprascapular nerve and vessels topography discovered in this case. In accordance with Tubbs et al.’s [20] statement, it can protect from neuropathy. Scientists hypothesize that a suprascapular notch that normally accommodates the suprascapular nerve may be less capacious if it also houses the suprascapular artery, which would exert pressure on the more fragile nerve. It may result in suprascapular nerve syndrome formation. In our opinion, superior bony bridge may also protect vessels from injury. On the other hand, the presence of inferior bony bridge decreases the total area of space for the traveling suprascapular nerve and vessels. Therefore, it might predispose to suprascapular nerve entrapment.

## Conclusions

A double suprascapular foramen is a very rare anatomical variant at the suprascapular region. Simultaneous ossification of STSL and the ACSL is proposed as the most probable explanatory mechanism of its formation. However, an alternative hypothesis based on the calcification of the bifid STSL is proposed. Knowledge of these potential anomalies and suprascapular nerve and vessels topography is essential for surgeons performing SN decompression, especially by means of arthroscopic techniques.
